# *Schistosoma* and *Leishmania*: An Untold Story of Coinfection

**DOI:** 10.3390/tropicalmed8080383

**Published:** 2023-07-27

**Authors:** Genil Mororó Araújo Camelo, Jeferson Kelvin Alves de Oliveira Silva, Stefan Michael Geiger, Maria Norma Melo, Deborah Aparecida Negrão-Corrêa

**Affiliations:** Department of Parasitology, Institute of Biological Sciences, Federal University of Minas Gerais, Belo Horizonte 31270-901, Brazil; genilmororo@gmail.com (G.M.A.C.);

**Keywords:** schistosomiasis, leishmaniasis, neglected tropical diseases, coinfection, polyparasitism, comorbidity

## Abstract

A remarkable characteristic of infectious diseases classified as Neglected Tropical Diseases (NTDs) is the fact that they are mostly transmitted in tropical and subtropical regions with poor conditions of sanitation and low access to healthcare, which makes transmission areas more likely to overlap. Two of the most important NTDs, schistosomiasis and leishmaniasis, despite being caused by very different etiological agents, have their pathogenesis heavily associated with immune-mediated mechanisms, and *Schistosoma* spp. and *Leishmania* spp. have been shown to simultaneously infect humans. Still, the consequences of *Schistosoma–Leishmania* coinfections remain underexplored. As the inflammatory processes elicited by each one of these parasites can influence the other, several changes have been observed due to this coinfection in naturally infected humans, experimental models, and in vitro cell assays, including modifications in susceptibility to infection, pathogenesis, prognostic, and response to treatment. Herein, we review the current knowledge in *Schistosoma–Leishmania* coinfections in both human populations and experimental models, with special regard to how schistosomiasis affects tegumentary leishmaniasis, discuss future perspectives, and suggest a few steps to further improve our understanding in this model of parasite–host–parasite interaction.

## 1. Often Forgotten, Never Gone: Neglected Tropical Diseases

People turn a blind eye daily, and it is no different when it comes to public health issues. Frequently forgotten and away from the spotlight, Neglected Tropical Diseases (NTDs) impose a devastating burden on the most vulnerable, marginalized populations [1]. They are most prevalent in tropical areas and affect over 1.7 billion people every year, who are exposed to pathogens, as well as their vectors and reservoirs [2]. Several targets were set by the World Health Organization (WHO) to prevent, control, eliminate or eradicate NTDs worldwide in 2020 [3], but some were not achieved, leaving us now with both new and old challenges heading towards 2030 [1]. In addition, the COVID-19 pandemic posed an unexpected, complicating factor in controlling NTDs. Health authorities have shifted their attention towards COVID-19, which lead to severe disruptions in NTD activities, including the diversion of financial resources, reassignment of personnel, discontinuation of monitoring programs, delays in diagnosis and treatment, and the overall collapse of health systems [2,4]. Among the control programs impacted by the pandemic, the ones aimed at controlling schistosomiasis and leishmaniasis were potentially two of the most severely hindered [5,6,7,8].

Schistosomiasis, a parasitic disease due to the infection by the trematodes from the genus *Schistosoma*, is endemic to 78 countries and caused a burden of 2.5 million disability-adjusted life years (DALYs) in 2016, leaving roughly 241.3 million people in need of mass drug administration in 2020 [2,9]. This genus comprises several species that can infect humans. *S. mansoni* is the only one present in the Americas; *S. japonicum* and *S. mekongi* affect parts of East and Southeast Asia; and Western Asia and Africa are afflicted by *S. mansoni*, *S. haematobium*, and *S. intercalatum*, with Sub-Saharan Africa being the most burdened [10].

Several clinical manifestations are attributed to schistosomiasis and, as an immunopathology, most of its symptoms are a side effect of the host’s own immune response. Although the granulomatous reaction is of utmost importance to contain toxic antigens secreted by trapped schistosome eggs, they are also the main cause of chronic pathology in this infection [11,12]. *S. mansoni*, *S. intercalatum*, *S. mekongi*, and *S. japonicum* infections mainly affect the intestines, liver, and spleen, while schistosomiasis caused by *S. haematobium* mostly compromises the urogenital system [13,14].

Leishmaniasis, in turn, is a group of diseases caused by protozoans from the genus *Leishmania*, divided into skin and systemic manifestations. Species such as *L. braziliensis*, *L. amazonensis*, *L. major*, and *L. tropica* mostly cause tegumentary leishmaniasis, including cutaneous and mucosal manifestations of leishmaniasis, while *L. infantum* and *L. donovani* are causative agents of visceral leishmaniasis [15,16,17,18,19,20,21]. Additionally, cases of post-kala-azar dermal leishmaniasis, a cutaneous manifestation, are reported after visceral leishmaniasis by *L. donovani* [15,16].

Tegumentary leishmaniasis is endemic to 89 countries and exhibited 221,953 cases in 2021, 99% of which happened in the Eastern Mediterranean, Argelia, and the Americas together [1,22,23]. However, its actual incidence has been estimated to be 2.5 to 4.3 times higher than what is reported [24]. Its clinical presentations affect the host’s integument, varying widely and being related to both the *Leishmania* spp. and the host’s immune response, ranging from self-healing localized ulcers to highly disfiguring mucosal lesions, due to extensive immune-driven tissue destruction, or diffuse nodular lesions, due to an anergic response [15,16,25].

Visceral leishmaniasis, on the other hand, is endemic to 80 countries and exhibited 11,743 cases in 2021, with three eco-epidemiological hotspots, East Africa, the Indian Subcontinent, and Brazil, reporting 94% of the cases that year [1,22,23]. In addition, although less prevalent than tegumentary leishmaniasis, this form is exceptionally lethal, leading to death in 95% of untreated symptomatic cases [1,24]. It mainly compromises internal organs such as the liver, spleen, and bone marrow, causing fever, anemia, hepatosplenomegaly, cachexia, and immunosuppression [16]. Pathogenesis has been related to parasite persistence, and an altered balance in different types of immune response [26,27].

As we resume activities aimed at controlling NTDs, we must bear in mind that the biological, geographical, and social contexts shared by several NTDs expose affected populations to a vast number of pathogens at the same time [1,2]. Therefore, it is reasonable to consider that a significant number of individuals in such areas are subjected to coinfections [28,29,30]. In addition, since the outcome of schistosomiasis and leishmaniasis is closely associated with the type and intensity of the host’s immune response during infection, it stands to reason that the immune response during coinfections may be altered to the extent that the prognosis of the diseases is modified [28,29,30]. Herein, we discuss the current knowledge on *Schistosoma–Leishmania* coinfections, an often-overlooked association, which may have profound consequences for the morbidity and treatment of either parasitic infection.

## 2. Two against the Host: The Influence of Schistosomiasis and Leishmaniasis on One Another

Coinfections with different species are a possibility worldwide as several human-infecting species of *Schistosoma* and *Leishmania* have distinct transmission areas. Coinfections with *S. mansoni* would be the only ones present in the Americas, while this and other species, e.g., *S. haematobium*, could be involved in coinfections in other regions, such as Africa and Western Asia [10]. Until now, only coinfections with *S. mansoni* and *Leishmania* spp. have been demonstrated in humans, mostly in Brazil [31,32,33,34] but also in Israel [35]. In this topic, we will explore what we know regarding the consequences of *Schistosoma–Leishmania* coinfections human populations.

The potential of schistosomiasis to interfere with other inflammatory diseases in humans, such as allergies, inflammatory bowel disease, toxoplasmosis, and malaria [36,37,38], has been investigated over the years, although not as extensively as in experimental models. However, much is left to uncover regarding coinfections with *Leishmania* spp.

Schistosomiasis seems to be detrimental to the treatment of tegumentary leishmaniasis. In the rural community of Corte de Pedra, Bahia, an endemic region in Northeastern Brazil, 88.3% of the people with active American tegumentary leishmaniasis (ATL) had helminth coinfections, with 16.7% being coinfected with *S. mansoni* [31]. The coinfected patients had an unsatisfactory response to antimonial therapy when compared with helminth-free individuals, with a higher frequency of persistent lesions, strongly associating helminth coinfections with a delayed lesion resolution [31]. A following randomized, double-blind, placebo-controlled trial in the same area found that early anthelminthic treatment did not improve on the subsequent antileishmanial therapy of *L. braziliensis*-coinfected patients [39]. In the State of Rio de Janeiro, Southeastern Brazil, 10% of the patients with American tegumentary leishmaniasis were infected with *S. mansoni*, and a higher frequency of therapeutic failure and relapse in helminth–*Leishmania* coinfected individuals was found as well [34]. Moreover, in a more recent case report, a coinfected patient from São Paulo, Southeastern Brazil, presented atypical manifestations of both schistosomiasis and ATL, and responded poorly to antimony treatment even after being treated with oxamniquine and praziquantel [33]. As indicated by O’Neal et al. [31], the helminth–ATL coinfected patients showed higher levels of serum immunoglobulin(Ig)E in comparison with ones with only ATL, indicating a higher interleukin(IL)-4 production in coinfected individuals that would contribute to these negative outcomes of hosts when faced with multiple infections.

**Figure 1:**
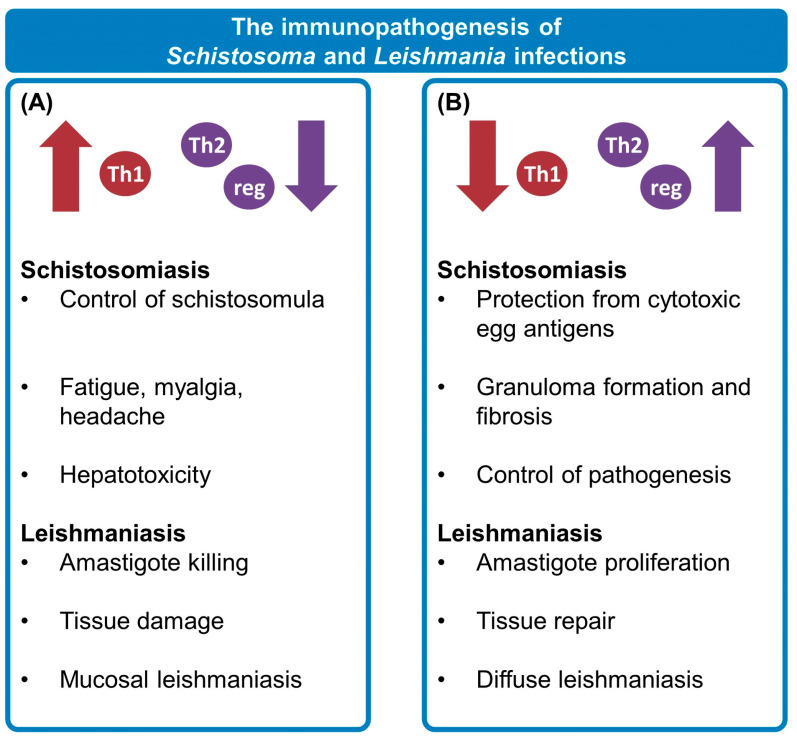
The immunopathogenesis of *Schistosoma* and *Leishmania* infections. The outcomes of both schistosomiasis and tegumentary leishmaniasis are heavily impacted by the host’s immune response, which is the main cause of pathology in these diseases. In the first weeks post-*Schistosoma* sp. infection, known as the pre-postural phase, a predominantly type 1 immune profile (**A**) is elicited in response to worm migration and maturation, participating in the control of schistosomula but also causing symptoms such as fever, fatigue, myalgia, and headache. After oviposition begins, hepatotoxic antigens are secreted by schistosome eggs. In response to these antigens, there is a change to a type 2 profile (**B**). This leads to circumoval granuloma formation, indispensable to properly containing cytotoxic egg antigens and protecting the adjacent tissues from damage; however, granulomas also cause fibrosis and are the main cause of pathology in postural acute schistosomiasis. Subsequently, a regulatory response (reg) takes over, reducing granuloma size and controlling pathology during the chronic phase of infection. On the other hand, these three profiles are highly associated with the outcome of tegumentary leishmaniasis, too, as type 1 responses (**A**) lead to amastigote killing but also damage neighboring tissue. An unchecked Th1 response is especially present in mucosal leishmaniasis. Meanwhile, type 2 and regulatory responses (**B**), important for tissue repair, favor amastigote proliferation, and the lack of an appropriate type 1 response can lead to anergy and diffuse leishmaniasis. Therefore, this fine balance between immune response profiles, parasite control, and tissue homeostasis may be considerably impacted during coinfections, possibly contributing to altered morbidity and response to treatment.

The immune responses elicited by both parasites, summarized in Figure 1, are rather complex. During *S. mansoni* development in the vertebrate host, a predominant type 1 response is stimulated against cercariae penetration, schistosomula migration, and adult worms [40,41,42], followed mostly by a type 2 profile after oviposition begins [43,44,45,46,47]. In parallel, a regulatory response is developed, which takes over in the chronic phase of schistosomiasis [48,49,50,51]. In contrast, in tegumentary leishmaniasis, an exacerbated T helper(Th)1-polarized response is related to increased tissue destruction in mucosal leishmaniasis [52,53,54], while the lack of an appropriate type 1 response and the predominance of Th2 and regulatory mechanisms lead to unchecked amastigote proliferation in diffuse cutaneous leishmaniasis [55,56,57,58]. Therefore, it is reasonable to expect that coinfected individuals could ultimately present a different disease outcome prevalence due to modified immune-mediated processes [28].

In vitro studies have shed some light on how immune response is altered in different *Schistosoma–Leishmania* coinfection models. The stimulation of peripheral blood mononuclear cells (PBMC) from ATL patients with soluble *Leishmania* antigens (SLA) in the presence or absence of different adult *S. mansoni* antigens has yielded interesting results [59,60]. Although each of the worm antigens generated different effects, the costimulation overall induced the production of IL-10, while inhibiting interferon(IFN)-γ [59]. Moreover, the presence of worm antigens decreased the expression of human leukocyte antigen(HLA)-DR, CD80, and CD86 in monocytes, while increasing the expression of cytotoxic T-lymphocyte-associated protein(CTLA)-4 in CD4^+^ T lymphocytes, and the frequency of CD4^+^CD25^high^FoxP3^+^ T cells [60]. Together, these results indicate that being exposed to adult *S. mansoni* antigens may hamper antigen presentation in ATL patients.

Similarly, by costimulating monocyte-derived dendritic cells (MoDCs) harvested from ATL patients with Sm29, an adult *S. mansoni* antigen, and SLA, the frequency of regulatory markers increased, while the populations of CD40^+^, IL-12^+^, and tumor necrosis factor(TNF)^+^ remained similar [61]. When co-cultures of MoDCs with autologous lymphocytes from patients with schistosomiasis were infected in vitro with *L. braziliensis*, a lack of activation and induction of the regulatory profile were also observed both in MoDCs and in CD4^+^ and CD8^+^ cells, which made MoDCs more susceptible to the in vitro infection [62]. Conversely, the stimulation of MoDCs from ATL patients with recombinant Sm29 did not increase their susceptibility to *L. braziliensis* in the presence of autologous lymphocytes in spite of the similarly induced regulatory profile [63]. Thus, even though the *S. mansoni* infection itself seems to increase host-cell susceptibility to *L. braziliensis*, schistosome antigens could potentially be used to decrease inflammation and aid the healing process during ATL [63].

Despite our current knowledge regarding the immune response to and pathogenesis of schistosomiasis and leishmaniasis separately, and what we may expect to change in a coinfection context, the immunopathology during human *Schistosoma–Leishmania* coinfections remains largely underexplored. The number and size of typical cutaneous lesions seem not to be affected regardless of helminth coinfections [31], but a case report describes a coinfected individual with unusual clinical presentations, with abdominal skin papules containing *S. mansoni* eggs, while *Leishmania* sp. lesions manifested as a crusted, infiltrated erythematous plaque with irregular borders [33]. Therefore, research on atypical manifestations of schistosomiasis and leishmaniasis in coinfected patients may be promising. Regarding the most severe forms of tegumentary leishmaniasis, and helminth coinfections, including *S. mansoni*, in ATL patients from Rio de Janeiro were shown to increase the prevalence of mucosal leishmaniasis [34]. However, it remains to be seen whether schistosomiasis itself could affect the incidence of mucosal or diffuse leishmaniasis.

Compared with tegumentary leishmaniasis, how schistosomiasis influences visceral leishmaniasis has not been addressed nearly as much. As pathogenesis in visceral leishmaniasis involves chemokine expression and the migration of immune cells in the liver and spleen [26,27], an infection with another parasite such as *S. mansoni*, which also alters cytokine profiles and cellular infiltration in these organs [47], is expected to heavily affect this process. In this context, it is worth mentioning a case report of enteropathic visceral leishmaniasis with concomitant *S. mansoni* infection, with persistent diarrhea, the presence of *L. infantum* amastigotes in duodenal biopsies, and relapses [64]. In addition, a case report of an Eritrean refugee in Israel suggests that *S. mansoni* in coinfection with a species from the *L. donovani* complex may exacerbate spleen-related pathology [35], but the amount of data we currently have is too small for anyone to imply that there is any causal relation. Furthermore, the fact that a case of *Schistosoma–Leishmania* coinfection has been diagnosed in Israel, a country not endemic for schistosomiasis, exemplifies that schistosomiasis and leishmaniasis are not only a problem in endemic countries. Therefore, policymaking in disease control should always take into consideration current migration processes.

In addition, the increased in vitro susceptibility to *Leishmania* sp. due to *S. mansoni* coinfection or the exposure to worm antigens may jeopardize the control of leishmaniasis. In another coinfection context, visceral leishmaniasis patients coinfected with the human immunodeficiency virus (HIV) can present high parasite loads in the skin and a higher frequency of post-kala-azar dermal leishmaniasis [65,66]. Therefore, they could potentially act like superspreaders due to the increased likelihood of infecting sand flies in addition to serving as reservoirs to drug-resistant *Leishmania* spp. [23,66]. It is not certain whether coinfections with *Schistosoma* spp. could pose the same threat or not since we currently do not know how *Schistosoma–Leishmania* coinfections influence the presence of amastigotes in the human tissues accessible by sand flies; however, the existing reports of diminished therapeutic efficacy in *Schistosoma–Leishmania* coinfections are becoming particularly worrying [31,33,39,64]. Therefore, in vivo peripheral parasite load must be addressed in forthcoming studies.

As for the impact of leishmaniasis on schistosomiasis in humans, we still know very little. Since schistosomula have been shown to be controlled by type 1 immune responses in the vertebrate host [40,41,42,67], the typical Th1 response elicited by *Leishmania* spp. infections [15,25,52] could theoretically favor the killing of immature stages in a *Schistosoma–Leishmania* coinfection setting. However, a study conducted by our group in an endemic region with the active transmission of *Leishmania* sp. and *S. mansoni* in Minas Gerais, Southeastern Brazil, showed that the prevalence of schistosomiasis was higher in individuals who presented a history of ATL [32]. Regarding the serum immune response, this condition was positively associated with an IL-27 response, while negatively associated with IL-17 and CXCL10, suggesting that a *Leishmania*-driven modulation could affect the host’s susceptibility to *S. mansoni* [32]. However, the most important take-home message from this study may be that simultaneous coinfection is not a requirement, as changes in the immune system caused by previous ATL still affect posterior *S. mansoni* susceptibility. Interestingly, this agrees with the fact that early anthelminthic treatment does not seem to improve the outcome of ATL [39], since helminth infections can also induce long-lasting effects on the host’s immune response [68,69,70,71,72]. Considering that people who live in coendemic areas may eventually come across these pathogens at different times, the lingering immunological shifts caused by the first infection could reverberate in future diseases for a yet-undetermined period of their lives. Thus, not only coinfections deserve attention, but non-concomitant parasite–immune system–parasite interactions do as well.

Despite the valuable knowledge provided by studies in human populations, some caveats must be pointed out. Up until now, the studies concerning the topic have been either cross-sectional or started after the onset of both infections, which makes it nearly impossible to determine the time and sequence of infections. Furthermore, other coinfections present in the studied populations, such as other helminthiasis and protozoan infections, might obscure the effect that a single species of parasite has. Therefore, some immunopathological aspects may not be easily explored in human populations. With that in mind, these limitations can be overcome with the use of experimental models, which provide a more homogenous, controlled environment that can yield new insights into the impacts and mechanisms involved in the *Schistosoma–Leishmania* coinfection.

## 3. Making Heads and Tails: Experimental Coinfection Models

Indeed, some questions raised from human studies have been better investigated with in vivo and ex vivo studies using mice, a host susceptible to infection with several *Leishmania* spp. and *S. mansoni* [25,73,74]. Although not extensive in number, these studies have slowly but steadily contributed to what we know about *Schistosoma–Leishmania* coinfections. Most published studies were performed using *L. major* and *S. mansoni*, with only a few exceptions, and some data still show contrasting results.

The experimental coinfection with *L. mexicana* in outbred mice that were previously infected by *S. mansoni* (60 days before) resulted in a shorter incubation period, with lesions appearing earlier in coinfected mice than in *L. mexicana*-monoinfected ones [75]. Meanwhile, C57BL/6 mice coinfected with *L. major* after 2 weeks of infection with *S. mansoni* presented larger lesions, but with delayed growth and resolution, which coincided with an impaired ability to control the parasite, resulting in higher *L. major* parasite loads [76]. Similarly, the concomitant infection of BALB/c mice with *S. mansoni* and *L. major* resulted in larger footpad lesions after 10 weeks and reduced mortality [77].

Popliteal lymph node cells harvested from C57BL/6 mice coinfected with *S. mansoni* and *L. major* also showed a different immune response after in vitro stimulation with SLA [76]. The results of La Flamme et al. [76] show that levels of IFN-γ, TNF-α, and nitric oxide (NO) were lower at 4 weeks of coinfection (6 weeks post *S. mansoni* infection, 4 weeks post *L. major* infection), with increased levels of IL-4. However, this profile was reversed from the 8th week post-coinfection onwards. Moreover, peritoneal macrophages from 6–8-week-schistosome-infected mice were unable to exert leishmanicidal activity in vitro when stimulated with IFN-γ, with a defective production of NO compared with macrophages from non-infected mice.

In contrast, no impact on lesion development kinetics was observed when the *L. major* infection happened 8 weeks after the *S. mansoni* infection or at the same time, with BALB/c mice developing chronic lesions, while C57BL/6 mice evolved to spontaneous healing [78,79]. In this case, cytokine measurement in popliteal lymph node cell culture by RT-PCR revealed that coinfected BALB/c mice expressed less IL-4 and more IL-10, while in C57BL/6 mice, the IFN-γ expression remained unchanged regardless of the coinfection, which is expected based on the clinical manifestations presented by both mouse strains [78].

These contrasting results may be due to differences in infective loads, the time of coinfection, the mouse strain, and the parasite species and strain, which are summarized in Table 1. A decisive factor in experimental *Schistosoma–Leishmania* coinfections seems to be the time of infection, as *L. major* coinfections that happen within the first weeks of the schistosome infection [76,77] were shown to alter the development of tegumentary leishmaniasis, while infection after 8 weeks of *S. mansoni* did not [78]. However, despite happening at approximately the same time after *S. mansoni* infection, coinfection with *L. major* [78] or *L. mexicana* [75] presented different outcomes. This only reaffirms the need to study different *Leishmania* spp., since the lack of changes in the pathogenesis or immune response by analyzing a single species should not be regarded as truth for others. Thus, studies regarding *Schistosoma* sp. coinfections with *L. braziliensis* and *L. amazonensis*, two of the main etiological agents of ATL [18,52,80,81], and viscerotropic *Leishmania* spp. are of utmost importance.

As for visceral leishmaniasis, it was found that *L. donovani* can thrive in both the liver and spleen of mice coinfected with *S. mansoni*. C57BL/6 mice infected with *L. donovani* 8 weeks after *S. mansoni* infection showed increased *L. donovani* parasite burdens in these organs, with the multiplication of amastigotes in egg granulomas associated with the lower production of NO and IFN-γ, and higher levels of IL-4 and IL-10 [82]. Additionally, once again, the impact of leishmaniasis on experimental schistosomiasis has been given less attention in comparison with the other way around. No changes in adult-worm load or eggs retained in the liver were observed in coinfected C57BL/6 mice when *L. major* infection happened 2 weeks after *S. mansoni* infection [76].

The impact of *Schistosoma–Leishmania* coinfections on the treatment success of schistosomiasis and leishmaniasis has also been investigated in experimental models. The combined treatment of *L. major*-and-*S. mansoni*-coinfected BALB/c mice using Pentostam and praziquantel was more effective than monotherapy in reducing skin lesions, Leishman-Donovan Units, and adult-*S. mansoni* recovery [79]. It is important to point out that it is unclear if the contrasting results regarding combined therapy in humans [39] and experimental models [79] is a product of different *Leishmania* spp. infections (*L. braziliensis* vs. *L. major*), a different leishmanicidal drug of choice (Glucantime vs. Pentostam), other coinfections (coinfection with several parasites vs. only *S. mansoni*), host-related factors or another variable. Thus, it may not be reasonable to directly compare these results, making it clear that more studies are needed to determine the best course of action when it comes to treating coinfected patients.

However, it is important to mention that experimental models also have their own caveats. Although mice and their several strains can reflect both self-healing and chronic skin lesions in tegumentary leishmaniasis [25], they may not replicate faithfully atypical or more severe forms of this disease, such as diffuse or mucosal leishmaniasis. Alternatively, metastatic forms of tegumentary leishmaniasis may be more closely evaluated by using trauma-induced metastasis [83]. In addition, Syrian golden hamsters (*Mesocricetus auratus*) are highly susceptible to both dermotropic and viscerotropic *Leishmania* spp., manifesting lesions similar to those in humans [84,85,86]. Other non-murine models, namely dogs, even though useful for replicating mucocutaneous disease [87], may not be appropriate for experimental *S. mansoni* infections [88].

As for *Schistosoma* spp., *S. mansoni* can infect mice and golden hamsters [73,74,89]. Regarding other models of coinfection, golden hamsters have also been used for *S. mansoni*–*Entamoeba histolytica* coinfection studies [90]. The limitations become more troubling when we consider that we still lack an accessible experimental model for *S. haematobium* infections, the etiologic agent of urogenital schistosomiasis. Rats, guinea pigs, and rabbits are not suitable hosts for this parasite, while mice and golden hamsters are only partially susceptible [73,74,91]. Although *S. haematobium* completes its life cycle in these two rodents, urinary involvement is absent in mice and inconsistent in hamsters [91]. Therefore, *S. haematobium–Leishmania* coinfections would be mostly limited to human studies and non-rodent models, which may further postpone advances in understanding changes in immunopathology during this coinfection and unraveling the involved mechanisms. This would not apply to *S. japonicum* and *S. intercalatum*, as they successfully infect mice [73].

A couple of unusual experimental models could be an alternative to work around some of the limitations presented by the more common rodent models. Some wild rodents, such as *Nectomys* spp. and *Holochilus* spp., can be maintained in laboratory and have been shown to be susceptible to *Leishmania* spp. and *S. mansoni* infections, and can also be found naturally infected [92,93,94,95,96]. Finally, while requiring a more sophisticated, expensive structure to maintain when compared with murine models, non-human primates might actually be a rather suitable model for the study of *Schistosoma–Leishmania* coinfections. First, several non-human primates are susceptible to both *Leishmania* spp. and *Schistosoma* spp. Additionally, *L. braziliensis* not only can induce chronic lesions in rhesus monkeys (*Macaca mulatta)* but they can also develop metastatic skin and mucosal lesions [97], providing a model for mucosal leishmaniasis under a *Schistosoma*-coinfection context. Diffuse leishmaniasis, as far as we know, has yet to be observed in non-human primates; however, it has been shown that *M. mulatta* is susceptible to infections with *L. amazonensis*, the main etiological agent of this clinical manifestation [18,98]. As for schistosomiasis, non-human primates, such as *Cercopithecus* spp., are susceptible to *S. mansoni* and *S. haematobium* infections both naturally and in a laboratory setting [99,100,101]. Moreover, the experimental infection of *C. fuliginosus* with *S. haematobium* more closely mimics human urogenital disease, with adult worms being present in prostatic, vesical, and uterine plexuses, with a high deposition of eggs in the bladder and uterus [101]. Therefore, these unusual models could be considered for studies on *Schistosoma–Leishmania* coinfections, especially when typical murine models are not appropriate.

## 4. Concluding Remarks and Future Directions

Even though it is not frequently given proper attention, *Schistosoma–Leishmania* coinfections could be more common than one would expect. Thus, this should be kept in mind when establishing control measures for both schistosomiasis and leishmaniasis. However, in the event that this coinfection becomes a reality when organizing control strategies for these diseases, much is still left to be uncovered.

We have yet to assess how widespread and numerous *Schistosoma–Leishmania* coinfections really are in different parts of the globe. Local studies that correlate the incidences of schistosomiasis and leishmaniasis can indicate areas where coinfections are more likely to occur, pointing research groups and authorities towards populations with a higher chance of presenting coinfected individuals. Longitudinal studies, on the other hand, provide an important tool in controlling the order of infection in endemic areas. In addition, the impact that this coinfection has on the transmission, burden, and treatment of such diseases must be better understood and would be a valuable aid in decision-making.

Studies using different *Leishmania* and *Schistosoma* species are essential, as, based on our current knowledge, interactions are likely different depending on the parasite species. Experimental coinfections could allow us to assess not only how the pathogenesis and parasite load change when different species are involved but also could provide a model which we can use to evaluate if *Schistosoma–Leishmania* coinfections can affect the transmission of these parasites, especially their infectivity to sand flies. Moreover, a better understanding of the immune mechanisms involved in this process could not only pave the way for more successful interventions in coinfected individuals but also potentially provide a better understanding of each infection separately.

## Figures and Tables

**Table 1:** Experimental design of *S. mansoni* coinfections with dermotropic *Leishmania* spp.

Reference	Mouse Strain	*S. mansoni* Strain	*S. mansoni* Load	*Leishmania* sp. and Strain	*Leishmania* sp. Load	Time of Coinfection	Changes in Skin Lesions
Coelho et al. 1980 [75]	Outbred mice	LE strain	70 cercariae	*L. mexicana* (L v 22)	1 × 10^8^ promastigotes	8 weeks post *S. mansoni* infection	Shorter incubation period
Yoshida et al. 1999 [78]	BALB/c & C57BL/6	Puerto Rico strain	20 cercariae	*L. major* (5ASKH)	4 × 10^7^ promastigotes	8 weeks post *S. mansoni* infection	Absent
la Flamme et al. 2002 [76]	C57BL/6	Puerto Rico strain	70 cercariae	*L. major* (FN)	5 × 10^6^ promastigotes	2 weeks post *S. mansoni* infection	Larger lesions with delayed healing
Yole et al. 2007 [77]	C57BL/6	Kenyan isolate	150 cercariae	*L. major* (NLB-144)	3 × 10^6^ promastigotes	Simultaneous	Larger lesions
Khayeka-Wandabwa et al. 2013 [79]	BALB/c	KEN-lab strain	70 cercariae	*L. major* (NLB-144)	1 × 10^6^ promastigotes	Simultaneous	Absent

## Data Availability

Not applicable.
